# Supplementary material for: Valorisation of Side Stream Products through Green Approaches: The Rapeseed Meal Case
Source: Foods. 2023 Sep 1;12(17):3286. doi: 10.3390/foods12173286 (PMC10486371; doi:10.3390/foods12173286)
Supplement: Supplementary file 1 [file foods-12-03286-s001.zip › foods-2538979-supplementary.pdf]

Valorisation of Side Stream Products through Green Approaches: The Rapeseed Meal Case

Francesco Cairone [1], Dario Allevi [2,3], Stefania Cesa [1], Giancarlo Fabrizi [1], Antonella Goggiamani [1], Domiziana Masci [2,3] and Antonia Iazzetti [2,3,*]

[1] Dipartimento di Chimica e Tecnologie del Farmaco, Sapienza, Università di Roma, P.le A. Moro 5, 00185 Rome, Italy
[2] Dipartimento di Scienze Biotecnologiche di Base, Cliniche Intensivologiche e Perioperatorie, Università Cattolica del Sacro Cuore, L.go Francesco Vito 1, 00168 Rome, Italy
[3] Policlinico Universitario 'A. Gemelli' Foundation-IRCCS, 00168 Rome, Italy
* Correspondence: antonia.iazzetti@unicatt.it

Table S1. Calibration curve of selective standards

	Retention time	Calibration curve (µg/mL)	Correlation coefficient
Gallic acid	4.86	y = 15.51x + 37.06	0.9987
Alliin	6.89	y = 6.35x + 50.34	0.9987
Catechin	15.48	y = 5.18 x - 24.29	0.9961
Chlorogenic acid	18.01	y =12.02x - 3.95	0.9991
Caffeic acid	19.01	y = 35.23x – 28.86	0.9989
Epicatechin	20.98	y = 2.47x + 58.32	0.9982
p-Coumaric acid	24.71	y = 42.12x – 19.25	0.9987
Cyanidin-3-rut	27.01	y = 16.58x + 34.53	0.9987
Ferulic acid	27.38	y = 20.65x + 22.96	0.9993
Sinapic acid	27.91	y = 11.37x + 9.92	0.9987
Myricetin	32.40	y = 21,51x – 5.93	0.9991
Quercetin	37.78	y = 21.69x + 24.12	0.9995
Kaempferol	42.25	y = 25.94x + 27.50	0.9988

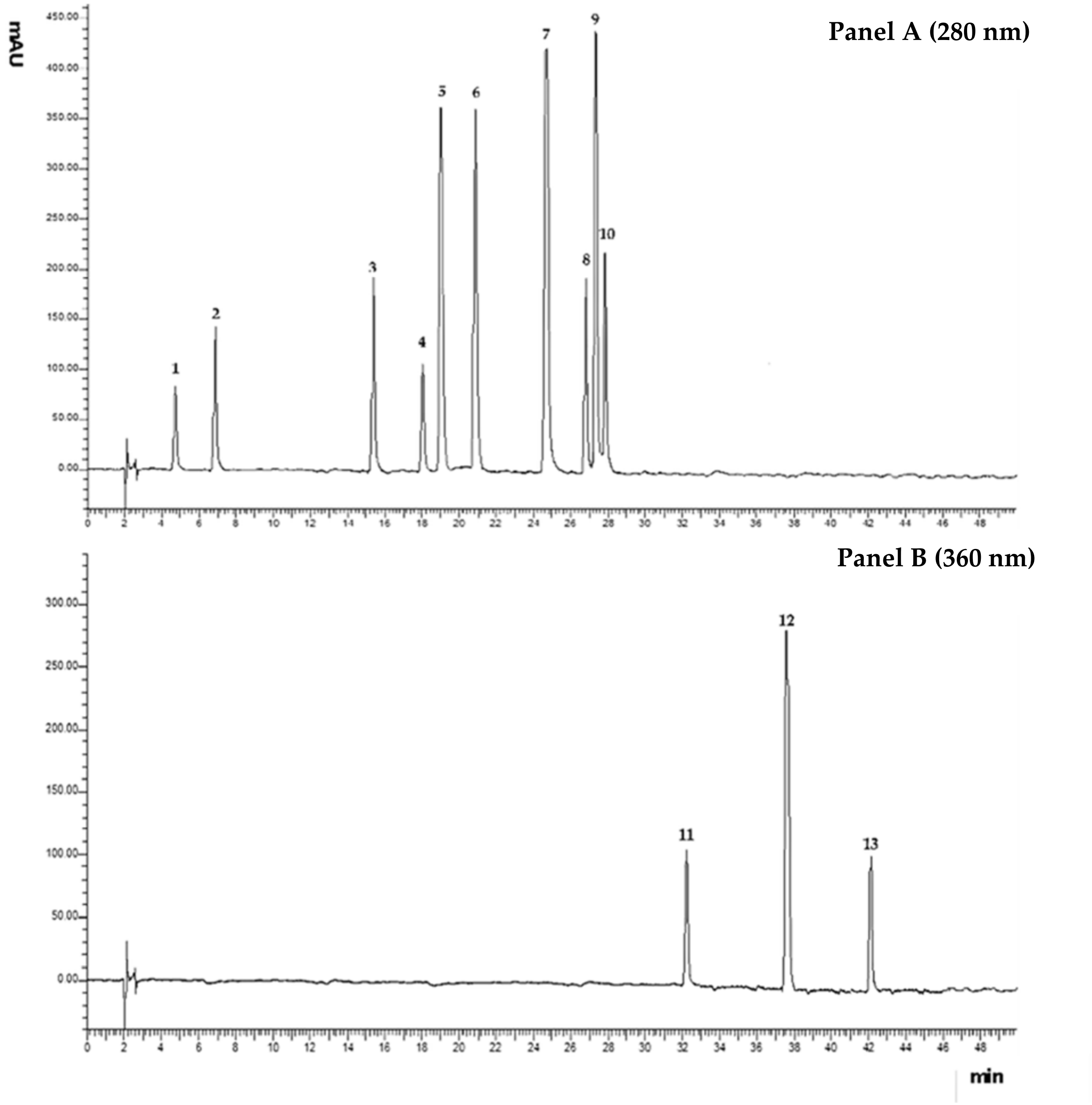

Figure S1. Chromatograms of standard compounds. Panel A 1. Gallic acid; 2. Alliin; 3. Catechin; 4. Chlorogenic acid; 5. Caffeic acid; 6. Epicatechin; 7. p-Coumaric acid; 8. Cyanidin-3-rutinoside; 9. Ferulic acid; 10. Sinapic acid; Panel B 11. Myricetin; 12. Quercetin; 13. Kaempferol.

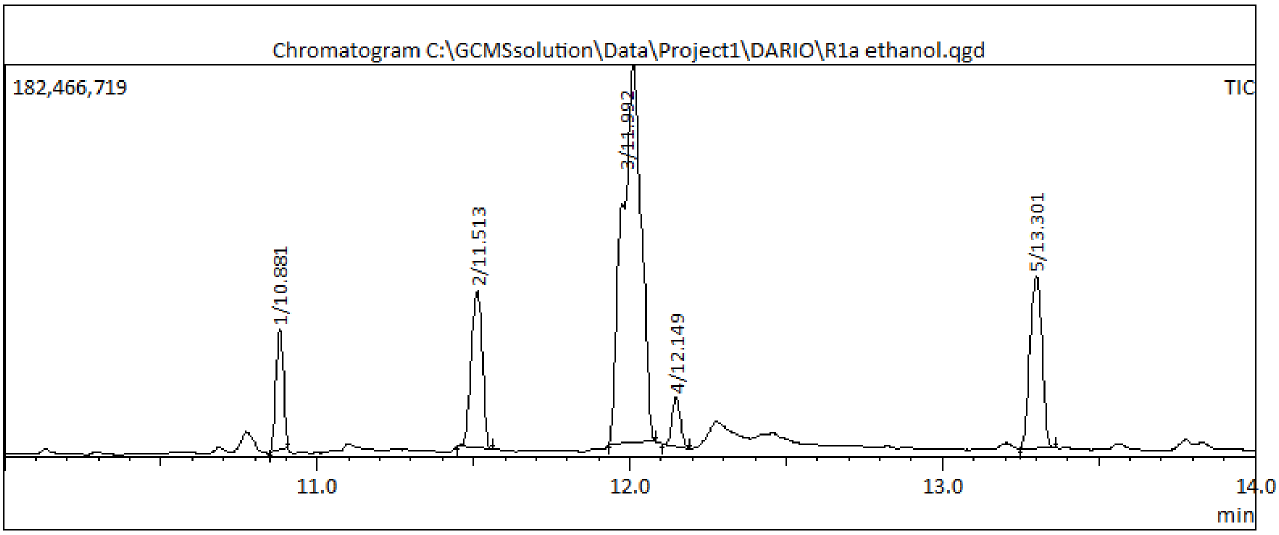

Peak Report TIC

Peak#	R.Time	I.Time	F.Time	Area	Area%	Name
1	10.881	10.847	10.907	92297344	7.24	
2	11.513	11.450	11.563	178259951	13.99	
3	11.992	11.933	12.080	746532945	58.58	
4	12.149	12.103	12.193	40702984	3.19	
5	13.301	13.247	13.360	216593267	17.00	
				1274386491	100.00	

Figure S2. Chromatogram and relevant peaks of GC/MS analysis of the methylated **R1a** residue.

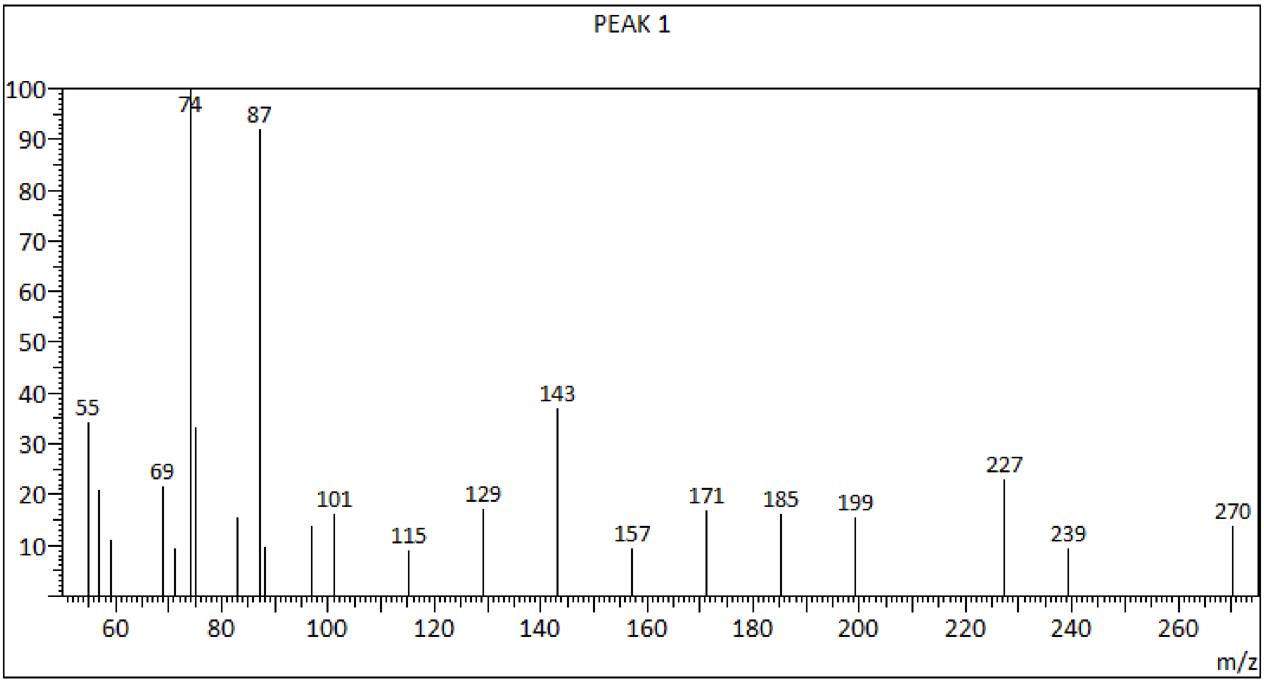

Figure S3. Mass spectrum for peak 1 (rt 10.881). The spectrum is consistent with the methyl esters of C16:0 (M+, m/z 270), **R1a** residue.

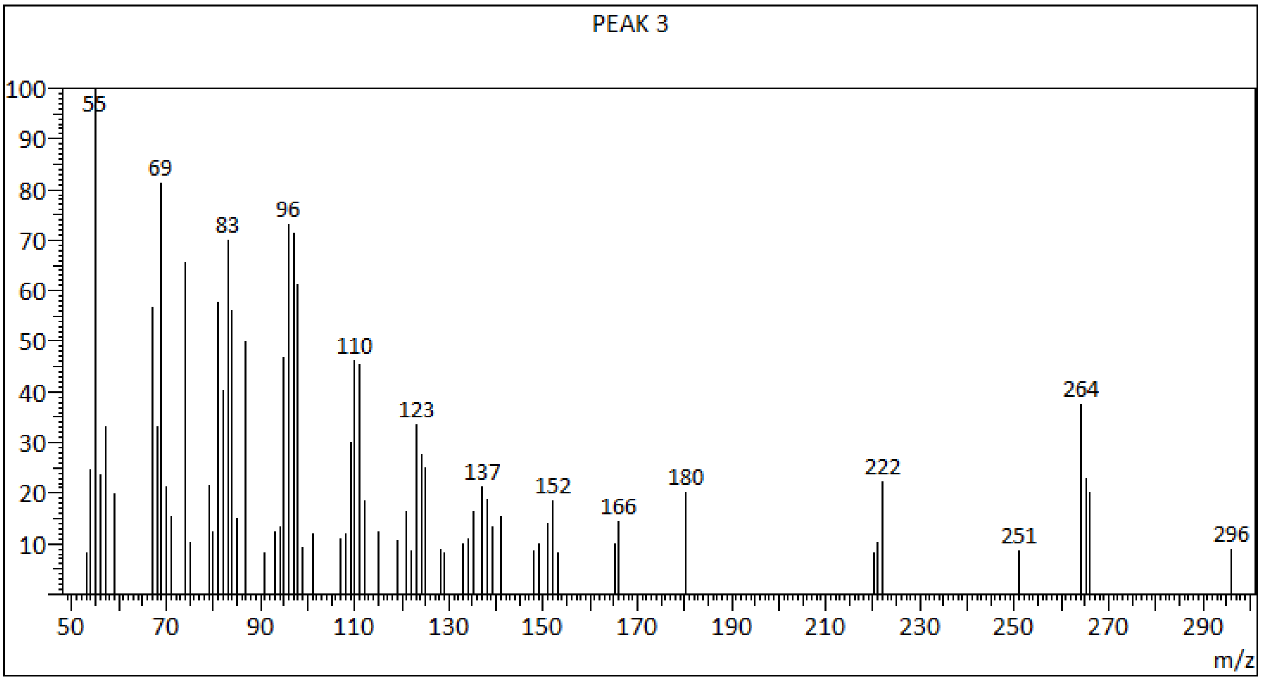

Figure S4. Mass spectrum for peak 3 (rt 11.992). The spectrum is consistent with the methyl esters of C18:1 (M+, m/z 296), **R1a** residue.

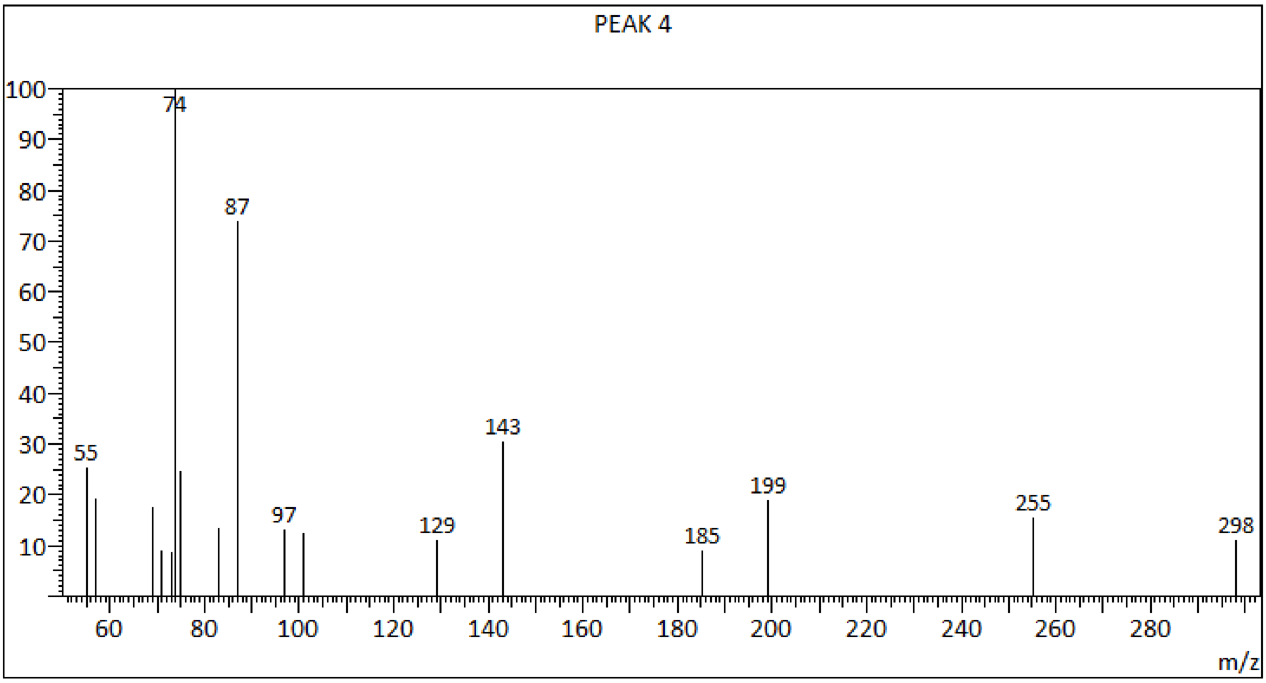

Figure S5. Mass spectrum for peak 4 (rt 12.149). The spectrum is consistent with the methyl esters of C18:0 (M+, m/z 298), **R1a** residue.

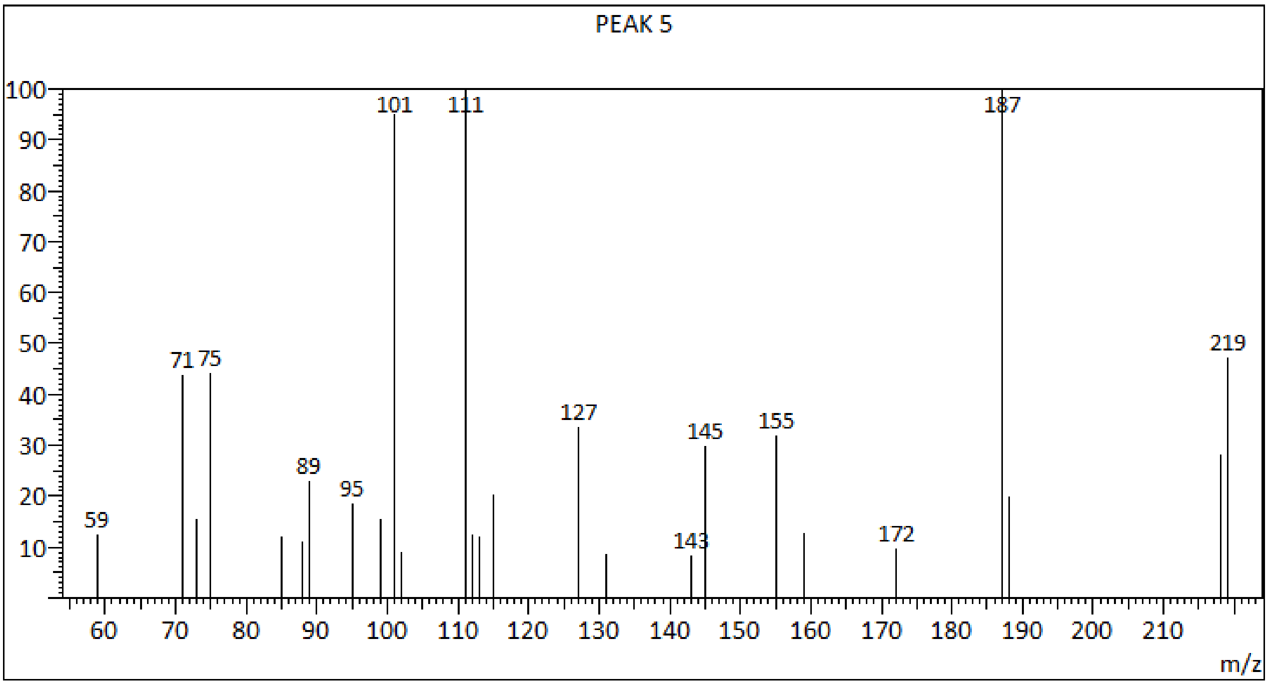

Figure S6. Mass spectrum for peak 5 (rt 13.301), **R1a** residue.

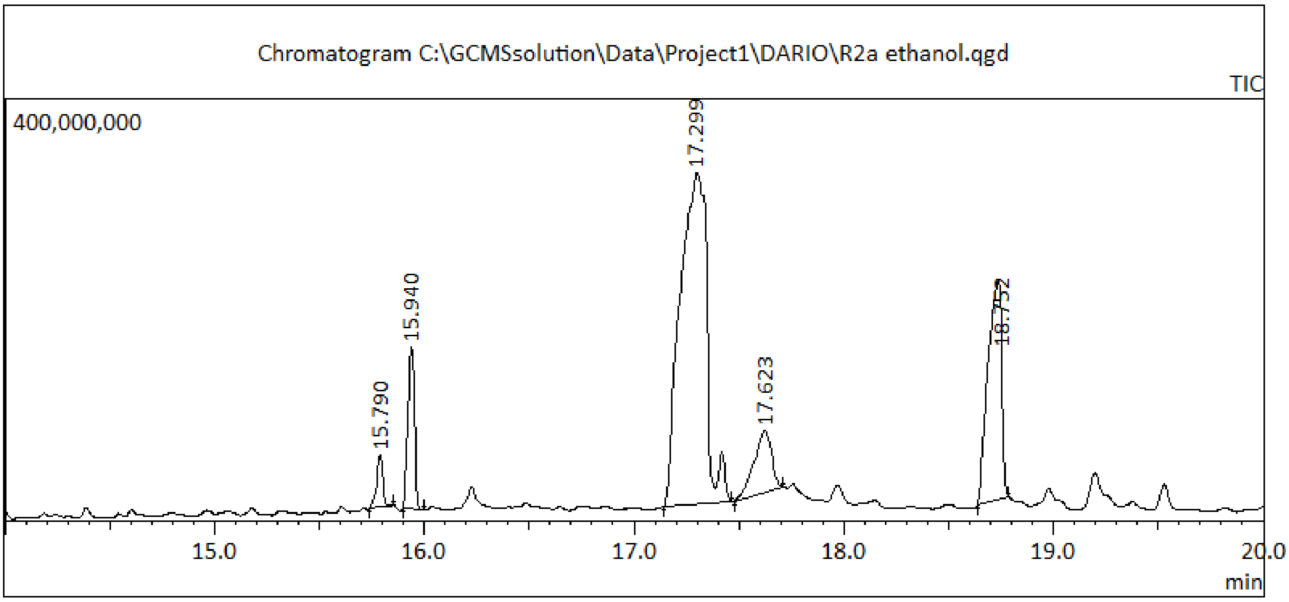

Peak Report TIC

Peak#	R.Time	I.Time	F.Time	Area	Area%	Name
1	15.790	15.740	15.850	93414199	2.07	
2	15.940	15.897	15.997	334137922	7.42	
3	17.299	17.143	17.463	2779950914	61.73	
4	17.623	17.477	17.713	343835340	7.63	
5	18.752	18.637	18.787	952242710	21.14	
				4503581085	100.00	

Figure S7. Chromatogram and relevant peaks of GC/MS analysis of the methylated **R2a** residue.

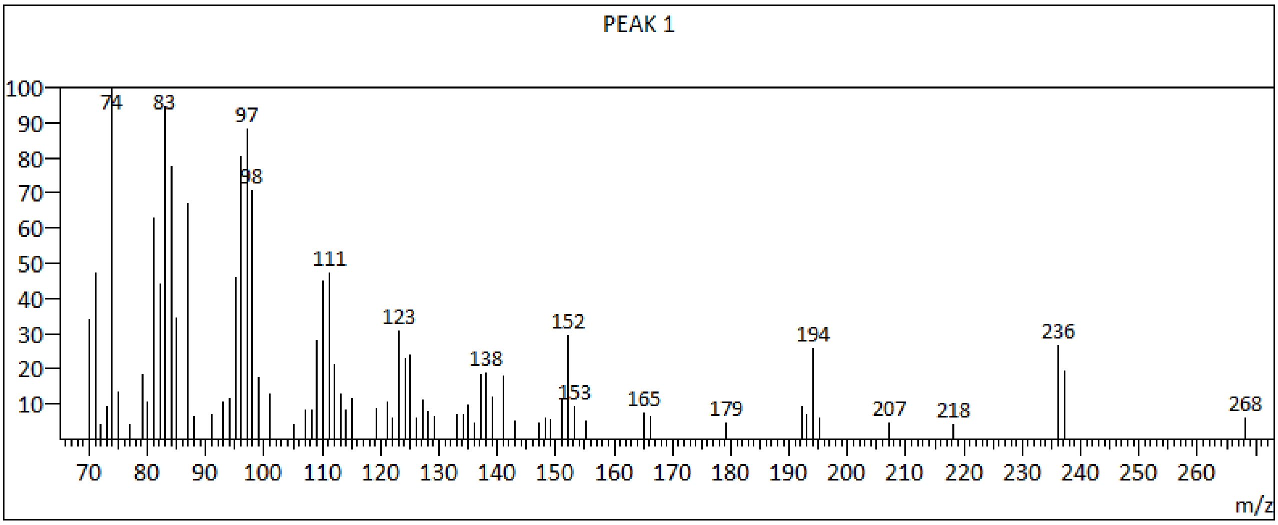

Figure S8. Mass spectrum for peak 1 (rt 15.790). The spectrum is consistent with the methyl esters of C 16:1 (M+, m/z 268), **R2a** residue.

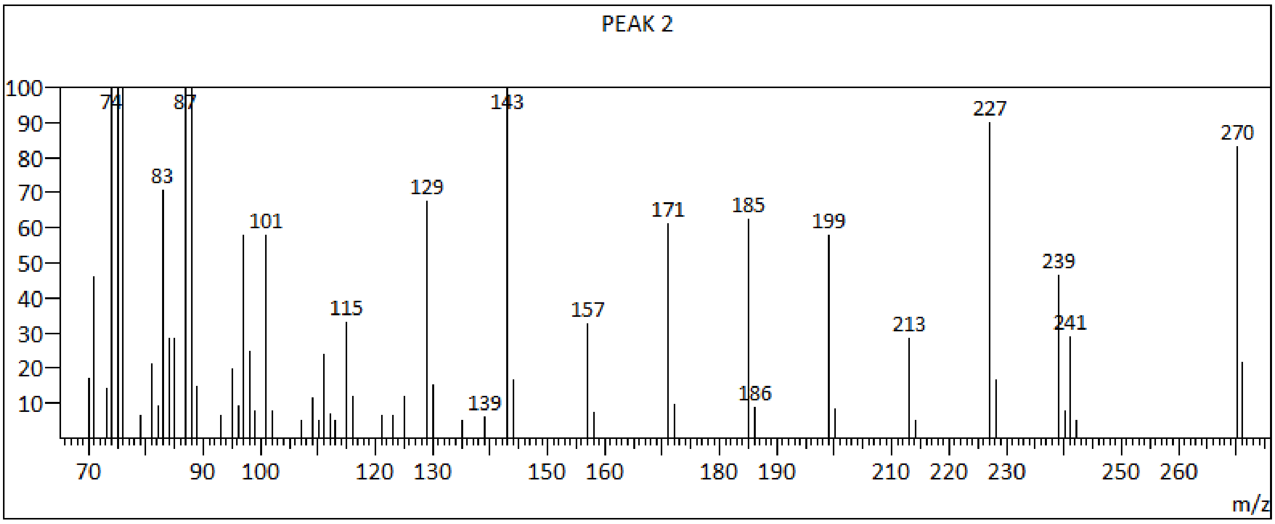

Figure S9. Mass spectrum for peak 2 (rt 15.940). The spectrum is consistent with the methyl esters of C 16:0 (M+, m/z 270), **R2a** residue.

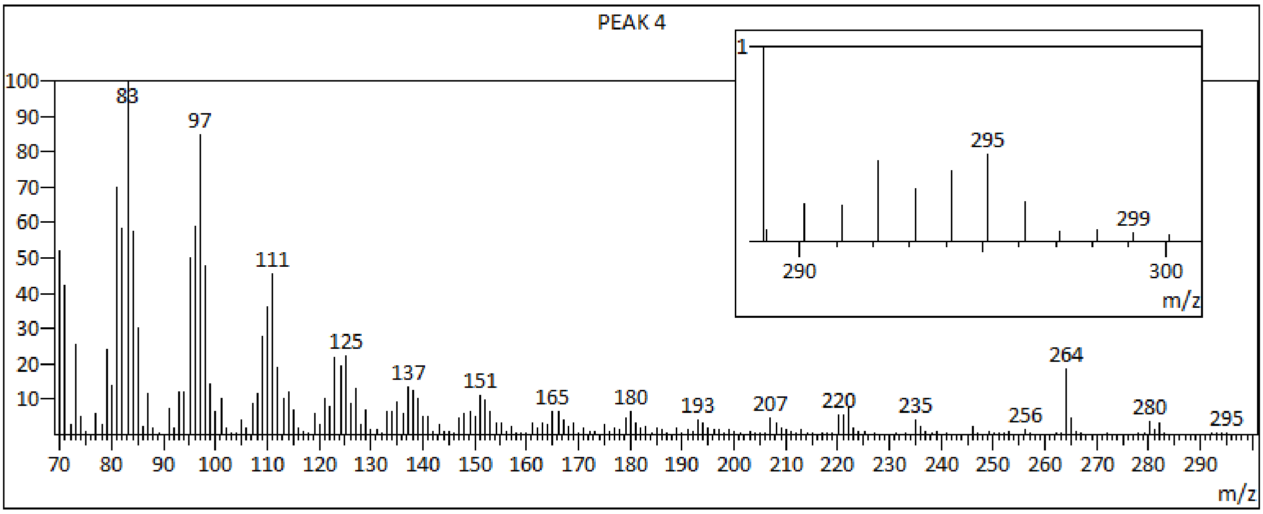

Figure S10. Mass spectrum for peak 4 (rt 17.623). The spectrum is consistent with the methyl esters of C 18:1 (M+, m/z 296), **R2a** residue.

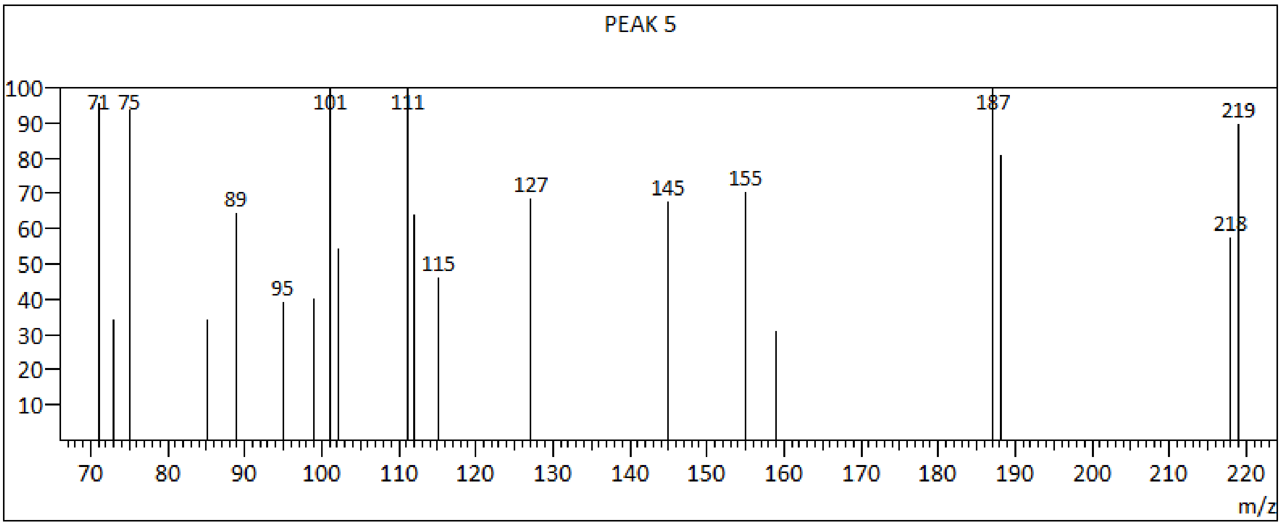

Figure S11. Mass spectrum for peak 5, **R2a** residue.
